# Seeing a straight line on a curved surface: decoupling of patterns from surfaces by single IT neurons

**DOI:** 10.1152/jn.00551.2016

**Published:** 2016-10-12

**Authors:** N. Apurva Ratan Murty, S. P. Arun

**Affiliations:** Centre for Neuroscience, Indian Institute of Science, Bangalore, India

**Keywords:** 3-D vision, object perception, object recognition, inferior temporal cortex

## Abstract

We have no difficulty seeing a straight line drawn on a paper even when the paper is bent, but this inference is in fact nontrivial. Doing so requires either matching local features or representing the pattern after factoring out the surface shape. Here we show that single neurons in the monkey inferior temporal (IT) cortex show invariant responses to patterns across rigid and nonrigid changes of surfaces. We recorded neuronal responses to stimuli in which the pattern and the surrounding surface were varied independently. In a subset of neurons, we found pattern-surface interactions that produced similar responses to stimuli across congruent pattern and surface transformations. These interactions produced systematic shifts in curvature tuning of patterns when overlaid on convex and flat surfaces. Our results show that surfaces are factored out of patterns by single neurons, thereby enabling complex perceptual inferences.

**NEW & NOTEWORTHY** We have no difficulty seeing a straight line on a curved piece of paper, but in fact, doing so requires decoupling the shape of the surface from the pattern itself. Here we report a novel form of invariance in the visual cortex: single neurons in monkey inferior temporal cortex respond similarly to congruent transformations of patterns and surfaces, in effect decoupling patterns from the surface on which they are overlaid.

## Abstract (precis)

We have no difficulty seeing a straight line on a curved piece of paper, but in fact, doing so requires decoupling the shape of the surface from the pattern itself. Here we report a novel form of invariance in the visual cortex: single neurons in monkey inferior temporal cortex respond similarly to congruent transformations of patterns and surfaces, in effect decoupling patterns from the surface on which they are overlaid.

we frequently recognize the same pattern overlaid on many types of surfaces (e.g., markings on animals, patterns on dresses, and text on banners). This is a nontrivial inference: it involves decoupling the pattern itself from the surface on which it is overlaid. This ability—which we term “surface invariance”—is a generalization of viewpoint invariance. In view invariance, a pattern on a surface can be recognized across rigid three-dimensional (3-D) rotations of the surface, whereas in surface invariance, a pattern can be recognized even across nonrigid transformations such as bending of the surface (Fig. 1*A*).

**Fig. 1.**
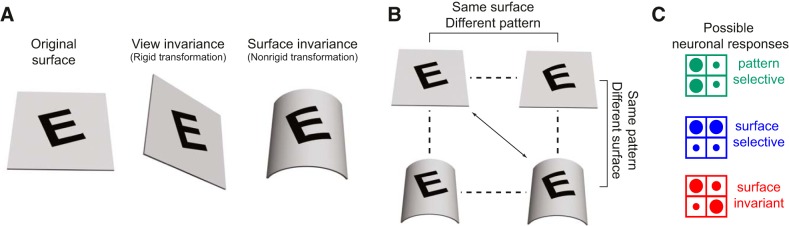
Surface invariance. *A*: view invariance represents our ability to recognize a pattern on a surface (*left*) after a rigid 3-D rotation (*middle*). We define as surface invariance our ability to recognize the pattern even after a nonrigid transformation (*right*). *B*: design of *experiment 1*. We independently varied the curvature of the pattern or surface to generate four possible stimuli. The stimuli along the main diagonal are equivalent across a nonrigid “bending” transformation. *C*: possible neuronal response patterns. At *top*, a neuron sensitive only to changes in the pattern would produce equal responses along columns (where the patterns are identical). In *middle*, a neuron sensitive only to changes in the surface would produce equal responses along rows (where the surfaces are identical). At *bottom*, a neuron that is surface invariant would produce equal responses to stimuli along the diagonal reflecting their equivalence across nonrigid transformations.

To investigate the neural basis of surface invariance, we targeted the monkey inferior temporal (IT) cortex, the terminus of the ventral visual pathway. IT neurons show invariant responses to objects across changes in position, size, and viewpoint (Brincat and Connor 2004; Hikosaka 1999; Hung et al. 2012; Ito et al. 1995; Yamane et al. 2008; Zoccolan et al. 2007). They are modulated by surface curvature (Brincat and Connor 2004; Yamane et al. 2008) and show nonlinear interactions between surface fragments (Brincat and Connor 2006). They are also selective for three-dimensional shape and show congruent selectivity for surfaces defined by texture or disparity (Liu 2004; Orban 2011). These properties make IT cortex a likely neural substrate for decoupling patterns from surfaces.

To investigate pattern and surface representations in IT, we designed tetrads of stimuli in which a pattern and a surface were manipulated independently to decouple their effect on the neuronal response. We have used the term “pattern” throughout because it implies two-dimensional features overlaid on a surface more than the term “shape” (which could be 3-dimensional). However, our results can be interpreted equivalently in terms of how surface shape is factored out of two-dimensional shapes overlaid on the surface. In the example stimulus set shown in Fig. 1*B*, the pattern (the letter E) is either flat or curved and is overlaid on a flat or convex surface. A neuron sensitive only to the pattern or the surface will produce similar responses along rows or columns (Fig. 1*C*). A cell that is surface invariant will respond similarly to stimuli along the main diagonal, which are related through a nonrigid bending operation (Fig. 1*C*). In contrast, a neuron sensitive to low-level pixel differences would show small changes in response along rows or columns (where only the pattern or surface change) and large response modulation along both diagonals (where both pattern and surface change). Thus sensitivity to low-level image changes cannot explain surface invariance.

Our results demonstrate that there is a subpopulation of surface-invariant IT neurons that decouple patterns across surfaces. These cells showed pattern-surface interactions that resulted in opposite tuning for pattern and surface changes. In a follow-up experiment, we found that these neurons show systematic shifts in curvature tuning for patterns juxtaposed against flat and curved surfaces. Taken together, our results suggest that IT neurons decouple patterns from surfaces to potentially enable complex perceptual inferences.

## METHODS

All experiments were performed in accordance with a protocol approved by the Institutional Animal Ethics Committee of the Indian Institute of Science, Bangalore, and the Committee for the Purpose of Control and Supervision of Experiments of Animals, Government of India. Many experimental procedures were similar to those described in a previous study (Ratan Murty and Arun 2015) and are therefore only summarized below.

### Neurophysiology

**Fig. 2.**
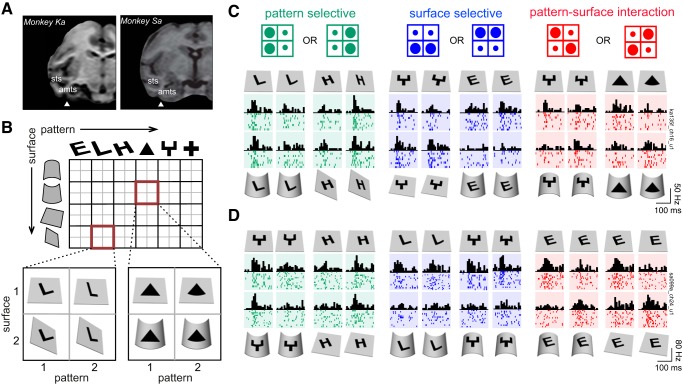
Example neuronal responses in *experiment 1*. *A*: structural MRI of both monkeys showing the approximate centers of recording locations in left IT (triangles). sts, Superior temporal sulcus; amts, anterior medial temporal sulcus. *B*: stimuli tested for each neuron. A total of six patterns were tested on four possible surface transformations (*top*). Each pattern-surface pair was used to create tetrads of four stimuli each (*bottom*). *C*: responses of an example IT neuron illustrating the diversity of effects observed in single neurons. This neuron showed main effects of pattern (green rasters), main effects of surface (blue rasters), and pattern-surface interactions (red rasters). Each row represents a single trial, and the black bars represent the firing rates in 20-ms bins. The dark-colored regions in each raster plot represent the stimulus duration (200 ms). It can be seen that the neuron responds similarly to stimuli along the main diagonal, particularly when it exhibits a pattern-surface interaction effect. Note that the pattern on the flat surface is common to all surface/pattern manipulations and therefore has identical responses. *D*: responses of a second IT neuron, with all conventions as in *C*. This neuron, too, showed main effects of pattern and surface in some cases and pattern-surface interactions for others. Importantly, as before, pattern-surface interactions were in the same direction whenever they occurred; that is, responses were similar to stimuli with congruent pattern and surface changes.

Two adult male monkeys (*Macaca radiata*, laboratory designations *monkeys Ka* and *Sa*, both aged 7 yr) were used in the study. Each animal was implanted with a titanium headpost and Cilux recording chamber (Crist Instruments). The recording chamber was centered on anterior left IT using structural MRI referenced to a standard rhesus monkey atlas (Saleem and Logothetis 2006). The location of the recording chamber was subsequently verified after placement using structural MRI. The center of the recording sites corresponded to anterior +14 mm and lateral +13 mm for *monkey Ka* and anterior +19 mm and lateral +15 mm for *monkey Sa*—which corresponded to anterior-ventral inferotemporal cortex (Fig. 2*A*). In *monkey Ka*, the recording chamber was positioned over anterior +19 mm but was tilted by 12° posteriorly and 7° laterally, making the effective recording location anterior +14 mm. Eye movements were monitored using an infrared eye tracker (ISCAN). Stimuli were displayed on a 120-Hz liquid-crystal display (LCD) screen (VX2268wm; ViewSonic). Stimulus presentation, eye position, and reward were under control of a computer running NIMH Cortex. On each day of recording, we inserted a multicontact electrode (Plexon 24 channel U-Probe with 100-μm interelectrode spacing along the shank) through a grid of 1-mm spacing, using a guide tube to ∼10 mm above IT cortex. We lowered the electrode further using a micromanipulator (Narashige) until phasic visual responses were observed. We isolated single units from the multiunit trace manually using commercially available spike-sorting software (Offline sorter V3; Plexon). To avoid instances when the same unit is picked up across multiple channels, we calculated a shuffle-corrected cross-correlogram for all pairs of simultaneously recorded channels and removed from further consideration units that had a cross-correlation peak exceeding 0.3 at zero lag. In all, our recorded population contained 106 well-isolated visually responsive neurons (62 from *monkey Ka* and 44 from *monkey Sa*) from 46 sites (29 from *monkey Ka* and 17 from *monkey Sa*) for *experiment 1*. For *experiment 2*, there were 58 visually responsive single units (34 from *monkey Ka* and 24 from *monkey Sa*) recorded across 38 sites (24 from *monkey Ka* and 14 from *monkey Sa*).

### Behavioral Task

Monkeys were trained to fixate a series of eight stimuli presented at the center of gaze (stimulus duration 200 ms followed by a 200-ms interstimulus interval) and received a juice reward for successfully maintaining fixation within a 3° fixation window. Post hoc analyses revealed that eye position was closely centered around fixation (average standard deviations across all trials and sessions: 0.22° about the vertical and 0.20° about the horizontal; we observed no systematic differences in gaze position across the 4 stimuli within a tetrad).

### Experiment 1: Decoupling of Patterns From Surfaces

#### Stimuli.

We tested a total of six patterns on four surfaces for each neuron. Each pattern-surface combination was used to create a set of four stimuli as depicted in Fig. 2*B*. Based on the rationale that IT neurons may encode patterns relative to the orientation or curvature of the surrounding surface, we tested four surfaces: two elemental curvatures (convex and concave) and two elemental viewpoint rotations (2-D rotation and 3-D tilt). The six patterns were chosen such that they contained no consistent alignment cues with the surfaces. Each pattern yielded four tetrads corresponding to each of the four surface transformations. However, since the pattern on the flat surface is common to the four tetrads, there were only 13 unique stimuli for each pattern, resulting in a total of 78 stimuli (6 patterns times 13 stimuli).

Stimuli were created using 3-D modeling software (Autodesk 3ds Max 2015; Academic License). Patterns (3.5° wide) were first imposed as textures on a flat surface (7° wide), after which the surface was transformed according to the surface type (convex/concave/rotated/tilted). Stimulus sizes were generally within the range of receptive field sizes reported for IT neurons (Connor et al. 2007; Tanaka 1992). However, we did not explicitly map the receptive field or tailor the stimuli to each neuron because our goal was to characterize how the neural population represents a fixed set of stimuli. However, note that the presence of a pattern-surface interaction implies that both the pattern and surface modulate the neural response, and therefore both must lie within the receptive field (see discussion). The three-dimensional shape of the surfaces was made apparent through shading cues (imposed using diffuse matte shading) and through the shape of the edges (tilted/curved). Incongruent stimuli were created by cropping out the central pattern from the flat and transformed surfaces and pasting them on the other surface using an image manipulation program (GIMP v2.8). All stimuli were presented as grayscale against a black background.

#### Trial design.

A total of eight stimuli were presented in a trial in pseudorandom order with the constraint that consecutive stimuli in a trial did not contain the same pattern or surface. Each stimulus was repeated at least eight times over the course of a recording session. Error trials were repeated after a random number of other trials.

### Experiment 2: Impact of Surface Curvature on Pattern Tuning

#### Stimuli.

For each neuron that appeared surface invariant in *experiment 1* during recording, we recorded responses to its preferred pattern by varying it parametrically from convex to flat to concave and overlaying these variations on either a convex or a flat surface (Fig. 8). The dimensions of stimuli were as in *experiment 1*. In some cases we recorded the response of the same neuron to other pattern variations as well, with the result that the data set contained 96 response sets collected from 58 neurons (34 from *monkey Ka* and 24 from *monkey Sa*).

Pattern curvature was varied by applying the flat pattern as texture on a flat surface and then transforming the surface in five equal steps in both concave and convex directions (30, 60, 90, 120, and 150° levels along each direction; “bend modifier,” Autodesk 3ds MAX). The resulting central patterns were cropped using GIMP and overlaid on a flat and a convex surface. The convex surface corresponded to a convex curvature of −3 levels away from flat (i.e., 90° level, “bend modifier,” Autodesk 3ds MAX). As a result, each curved pattern on a flat surface would be equivalent (through a nonrigid transformation) to a pattern shifted exactly by 3 levels on the convex series (Fig. 8). This yielded a total of 22 stimuli that were tested for each neuron (11 pattern curvatures against 2 surfaces). We tested each neuron also on concave surfaces, but we observed inconsistent interaction effects possibly because of sampling issues (see results).

#### Trial design.

All other details of the experiment were identical to *experiment 1*.

### Data Analysis

#### Pattern decoding.

**Fig. 3.**
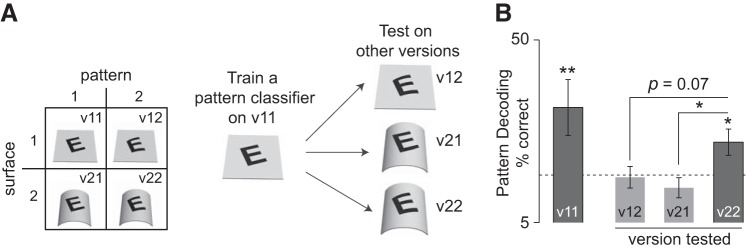
Surface-independent pattern decoding by the IT population. *A*: at *left*, schema indicating the four stimuli in a tetrad, labeled as v11, v12, v21, and v22. At *right*, schematic of the pattern decoding analysis: a pattern classifier was trained on decoding pattern identity on the flat surface (v11) and then tested on the other versions (v12, v21, and v22). *B*: accuracy of the pattern classifier trained on the patterns on the flat surface (v11, accuracy obtained using leave-one-out cross-validation) and tested on the other versions v12, v21, and v22. The dashed line represents chance performance (random guessing would yield 1 of 6 correct, i.e., 16%). Error bars indicate SE, and asterisks indicate statistical significance (*P* < 0.05, rank-sum test between the vector of correct and incorrect responses for each pair of versions).

For each trial of the stimulus, we calculated the population response vector with the firing rates (50–150-ms poststimulus onset), elicited by all 106 visual neurons. This analysis assumes that the responses were recorded simultaneously (which is not the case) and therefore can be considered as a lower bound on the information in the population (Hung et al. 2005). We then projected the entire set of responses along their principal components to retain most (70–80%) of the total variance. This step had the effect of removing cells with redundant and noisy responses (varying this fraction yielded qualitatively similar results). We then trained a multiclass linear discriminant classifier (“classify” function in MATLAB) on the responses and the corresponding six pattern labels for version v11 (Fig. 3*A*). We evaluated the performance of the classifier to decode the pattern on v11 using a leave-one-out approach: for each trial we trained the classifier on the remaining trials and predicted the pattern label. This yielded a percent-correct accuracy measure that represents how well pattern identity could be decoded from the neural population. To evaluate whether the pattern classifier trained on v11 generalized to the other versions, we tested it on the other versions (v12, v21, and v22). This yielded a percent correct measure that indicates how well a pattern classifier trained on flat surfaces (v11) can decode patterns across other versions (v12, v21, and v22) across all surface types.

#### Selection of tetrads with visual responses.

We estimated the spontaneous activity of each neuron in a 200–400-ms window after the end of the trial. The spontaneous activity was compared with the response to each stimulus in the tetrad in a window 0–200 ms after stimulus onset using a rank-sum test. Tetrads were included for further analysis if at least one of the four responses was statistically significant compared with spontaneous levels (α = 0.05, rank-sum test). Of the 2,544 tetrads (106 neurons times 6 patterns times 4 surfaces) tested in this manner, 1,608 tetrads (63%) were determined in this manner to be visually responsive to at least 1 of the stimuli. We obtained similar results using the spontaneous activity estimated from the pre-stimulus onset period.

#### Multidimensional scaling analysis.

To visualize the average similarity relations within a tetrad, we calculated the pairwise dissimilarity between stimuli as the average absolute difference in firing rates (50–250 ms after stimulus onset). In all, there were six pairwise distances across the four stimuli in each tetrad, which were averaged across all tetrads. We then performed a multidimensional scaling on the average distances between stimuli in tetrads of a particular type (e.g., those that contained interaction effects). This yielded a set of 2-D coordinates whose pairwise distances produce the best match between the six observed average pairwise distances. This was implemented using the “mdscale” function in MATLAB (MATLAB 2015a; MathWorks).

## RESULTS

To investigate the neural basis for how patterns are decoupled from surfaces, we recorded neuronal responses from the left anterior IT cortex (Fig. 2*A*) of two monkeys that viewed images during a fixation task. In *experiment 1*, we tested IT neurons on a number of patterns and surfaces to characterize the strength and extent of surface invariance. In *experiment 2*, we investigated whether surface-invariant responses arise from systematic shifts in pattern tuning due to modulation by the surrounding surface.

### Experiment 1: Decoupling of Patterns From Surfaces

In this experiment, we tested a total of 106 visually responsive IT neurons using a fixed set of 6 patterns and 4 surface transformations (Fig. 2*B*). Our rationale for selecting surfaces was that, to decouple patterns from surfaces in general, neurons would need to decouple patterns from elemental surface transformations such as bending or tilting. We therefore tested four surface transformations: bending of the flat surface to become convex or concave (i.e., nonrigid transformations) or tilting the flat surface either in plane or in depth (i.e., rigid/affine transformations). For each pattern-surface combination, we created four stimuli in which the pattern and surface varied independently (Fig. 2*B*). In this design, a neuron selective only to the central pattern will produce similar responses along columns (Fig. 1*C*, *top*). This would produce a main effect of pattern in an ANOVA on the firing rate with pattern and surface as factors. A neuron sensitive only to surface changes would produce similar responses along rows, respectively, which would represent a main effect of surface in the same ANOVA (Fig. 1*C*, *middle*). A neuron that decouples patterns from surfaces would produce similar responses along the main diagonal, which corresponds to an interaction effect (Fig. 1*C*, *bottom*). Note that similar responses along either diagonal would produce an interaction effect, but we are interested in a particular type of interaction that produces similar responses along the main diagonal (indicating stimuli equivalent across rigid and nonrigid transformations) but not the other diagonal.

The example responses of two IT neurons are shown in Fig. 2, *C* and *D*. These responses illustrate the considerable diversity we observed in our data. Both neurons showed pattern and surface main effects (Fig. 2, *C* and *D*, green and blue rasters). Notably, however, they showed similar responses to the congruent pattern/surface combinations, particularly when they show an interaction effect (Fig. 2, *C* and *D*, red rasters).

### Pattern Classification

Despite the diversity in the individual neuronal responses, the neuronal population as a whole may systematically signal patterns independent of surfaces. For ease of exposition, we denote the four stimuli in a tetrad as v11, v12, v21, and v22, where v11 is the flat pattern against the flat surface, v12 is the curved pattern on the flat surface, etc. (Fig. 3*A*). To investigate whether pattern decoding generalizes across surfaces, we trained a linear classifier to decode patterns overlaid on flat surfaces (i.e., on the v11 versions of all patterns) using all 106 visually responsive neurons. The performance of this classifier was well above chance (Fig. 3*B*), which is not surprising considering IT neurons are selective to these patterns. Critically, we next asked whether the same classifier, trained on patterns overlaid on flat surfaces, could correctly identify the pattern in the other versions (i.e., on the v12 versions of all 6 patterns, or v21 or v22 versions, etc.). The classifier showed an above-chance generalization only for the congruent version (v22) but not for the other versions (Fig. 3*B*), indicating that cells informative about pattern identity also generalize across rigid and nonrigid surface changes. Thus, IT neurons show surface-invariant pattern decoding at the level of the population.

### Incidence of Pattern, Surface, and Interaction Effects in IT

The above analysis shows that the IT neuronal population as a whole exhibits surface invariance, but these effects could be driven at the single-neuron level by a smaller subset of neurons that show systematic variations across the four stimuli in each tetrad. To understand these variations better, we analyzed each tetrad separately to assess how pattern, surface, and interaction effects were distributed (see below for neuronwise analyses). We performed an ANOVA on the firing rate of each neuron (in a 50–250-ms window after image onset) to the stimuli in each tetrad, with pattern (flat/transformed) and surface (flat/transformed) as factors. There were thus a total of 24 tetrads per neuron (6 patterns times 4 surfaces each), resulting in a total of 2,544 tetrads. Of the 2,544 tetrads, 1,608 of them (63%) elicited a significant visual response in at least 1 of the 4 stimuli in the tetrad (see methods) and were considered for further analysis.

**Fig. 4.**
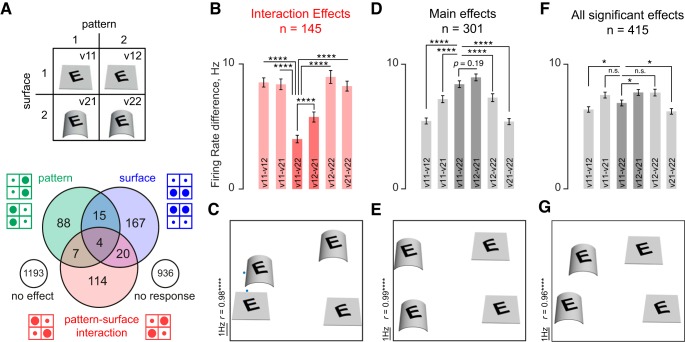
Surface invariance in IT neurons. *A*: we performed an ANOVA on the responses to each tetrad with pattern and surface as factors (*top*). The distribution of main and interaction effects across tetrads is shown below. A total of 2,544 tetrads were analyzed, of which 936 tetrads elicited no significant visual response to any of the stimuli. The numbers of tetrads with significant effects of pattern, surface, and pattern-surface interaction are shown as a Venn diagram. *B*: neuronal dissimilarity between stimuli across tetrads with interaction effects (*n* = 145), as measured using average absolute difference in firing rates. The stimuli along the main diagonal (v11–v22) were the most similar across these tetrads. Here and in the remaining figures, asterisks represent statistical significance on a Wilcoxon signed-rank test performed on the pairs of distances across the corresponding set of tetrads (*P < 0.05, **P < 0.005, etc.; n.s., not significant). *C*: visualization of neuronal dissimilarity relations in *B*, obtained using multidimensional scaling. Nearby images indicate similar responses. Most images are centered above their two-dimensional coordinates obtained from multidimensional scaling, but v11 and v22 are depicted beside their respective coordinates (cyan dots) to avoid overlap. It can be seen that the responses to congruent pattern-surface changes (v11–v22) are the most similar. The E on a convex surface is only shown as an example, but the distances are based on averaging across all surfaces and patterns that produced interaction effects. The correlation value indicates the match between the distance in the multidimensional scaling plot and the actual neuronal distances. *D*: neuronal dissimilarities between stimuli across tetrads with main effects of either pattern or surface (*n* = 301). *E*: visualization of neuronal dissimilarities in *D*. *F*: neuronal dissimilarities between stimuli across tetrads with either main or interaction effects (*n* = 415). *G*: visualization of neuronal dissimilarities in *F*.

The distribution of significant effects obtained in the ANOVA across the visual tetrads is shown in Fig. 4*A*. A majority of tetrads (1,193/1,608, i.e., 74%) did not show significant modulation to the pattern and surface changes, indicating equal responses to all four stimuli in the tetrad. The remaining tetrads showed significant response modulation to pattern, surface, or their interaction (*n* = 415 or 26%). These significant effects were observed across 86% (91/106) of all cells. Among these significant effects, nearly half (206/415 or 50%) showed a main effect of surface, a third of them (114/415 or 27%) showed a main effect of pattern, and a slightly larger fraction (145/415 or 35%) showed interaction effects. Although the interaction effects were relatively few in number across all visual tetrads (145/1,608 or 9%), this proportion was significantly greater than the fraction (5%) expected by chance (*P* < 0.00005, chi-squared test). Notably, these interactions were present in a majority of cells (70/106 or 66%) and were present more consistently in some cells than others (see below).

### Analysis of Surface-Pattern Interactions

The above analyses show that IT neurons are modulated by both pattern and surface curvature and also exhibit pattern-surface interactions. However, a pure pattern-surface interaction represents equal responses along each diagonal of a tetrad with one diagonal being larger than the other (Fig. 4*A*). We therefore asked whether these interactions resulted in a specific type of response in each tetrad. Of particular interest to us was whether the interactions resulted in similar responses to the two stimuli along the main diagonal, i.e., when both pattern and surface show congruent changes.

We measured the neuronal dissimilarity between two stimuli as the absolute difference between the responses to the stimuli averaged across the population (Sripati and Olson 2010b). We then averaged the neuronal dissimilarity between all six possible pairs of stimuli (4 choose 2) across tetrads with significant pattern-surface interactions (Fig. 4*B*). The firing rate difference along the main diagonal (v11–v22) was smaller than all other differences (average absolute firing rate differences: 8.5, 8.4, 4.0, 5.6, 9.0, and 8.2 Hz for v11–v12, v11–v21, v11–v22, v12–v21, v12–v22, and v21–v22, respectively; *P* < 0.00005 on a signed-rank test comparing v11–v22 difference with all other pairs across tetrads).

Note that by selecting interaction effects, we are by definition selecting responses that are stronger along one diagonal compared with another, but this selection does not predict which diagonal should show more similar responses. Thus the critical comparison is between the two diagonals which yielded a statistically significant difference. Likewise, if response patterns were idiosyncratic across neurons, we would have observed equal numbers of tetrads with similar responses along either diagonal. Thus the observed pattern of firing rate differences is nontrivial and cannot be due to idiosyncratic neural selectivity. Thus, among the tetrads with interactions, stimuli with congruent pattern and surface changes are most similar to each other compared with all other pairs. We obtained qualitatively similar results for both rigid (tilt/rotate) and nonrigid (convex/concave) surface changes (see below).

Although the above results directly show that pattern-surface interactions lead to similar responses to congruent pattern and surface changes, it is instructive to visualize these neuronal dissimilarities using multidimensional scaling. In the resulting plot, stimuli are placed far apart if their average neuronal dissimilarity was large. Across tetrads with interaction effects, it can be seen that the congruent pattern-surface pairs are closer to each other, reflecting their equivalence across both nonrigid and rigid transformations (Fig. 4*C*).

The surface-pattern interactions could have arisen if one of the off-diagonal stimuli (i.e., versions v12 or v21) produced a large response compared with all other stimuli. To test this possibility, we identified the stimulus with the largest response in each tetrad and asked whether the off-diagonal stimuli (v12 or v21) tended to be the best stimulus more often than expected by chance (50%). The off-diagonal stimuli elicited the largest response in 54% of the interaction tetrads (79 of 145) but this fraction was not significantly different from chance (*P* = 0.32, chi-squared test). We conclude that surface-pattern interactions do not arise due to isolated large responses to the off-diagonal stimuli within a tetrad.

The pattern-surface interaction effects could also have arisen because some patterns (triangle, Y, and +) contained edges that were aligned to the surface edges in the two congruent variations (v11 and v22). If this were so, it would predict greater pattern-surface interaction effects for these patterns compared with the others (E, L, and H), which were rotated away so as to preclude alignment cues. However, we did not see such a predisposition across the observed interaction effects: 47% (68 of 145) of interactions involved the aligned patterns (triangle, Y, and +) while 53% (77 of 145) of them involved the tilted patterns. These proportions were not significantly different from the 50-50 split expected by chance (*P* = 0.51, chi-squared test). We conclude that the observed surface-pattern interactions do not arise due to special alignment cues between the patterns and surfaces used in this experiment. Further support against this possibility comes from *experiment 2*, in which we find that neurons show systematic shifts in their pattern tuning when patterns are overlaid on flat or convex surfaces (see below).

The pattern-surface interactions observed may be more frequent for the preferred stimuli in each neuron. To assess this possibility, we sorted the responses to the six patterns tested for each neuron against a flat surface from low to high and asked whether pattern-surface interactions were more frequent for the three most preferred patterns compared with the least-preferred patterns. We observed no significant predisposition toward the preferred patterns compared with the 50-50 split expected by chance (number of interactions: 72 for preferred patterns, 73 for nonpreferred patterns, *P* = 1.00, chi-squared test).

### Analysis of Surface and Pattern Main Effects

We also performed similar analyses on the tetrads that exhibited at least one main effect (*n* = 301). The resulting pattern of neuronal similarity was qualitatively different (Fig. 4*D*). Although there was a still a tendency toward the firing rate difference along the main diagonal (v11–v22) to be smaller than along the other diagonal (v12–v21), this effect did not reach statistical significance (average firing rate difference: 8.5 and 8.9 Hz, respectively, *P* = 0.19 on a signed-rank test across 301 tetrads with main effects). Importantly, however, the smallest change in firing rate occurred for changes in pattern only (v11–v12 and v21–v22; Fig. 4*D*) and not for the congruent stimuli (i.e., for v11–v22). These similarity relations can be seen at a glance in the multidimensional scaling plot (Fig. 4*E*).

Finally, we considered the overall neuronal similarity across the entire population of tetrads with significant main or interaction effects. This too revealed a pattern similar to the main effects (Fig. 4*F*): pattern changes caused the smallest change in firing rate, but there was a significant tendency for the stimuli along the main diagonal to be more similar than the other diagonal (average firing rate difference: 7.0 and 7.7 Hz, respectively, *P* < 0.05 on a rank-sum test across 415 tetrads). These similarity relations can be seen visually in the multidimensional scaling plot (Fig. 4*G*).

### Analysis of Neuronwise Effects

**Fig. 5.**
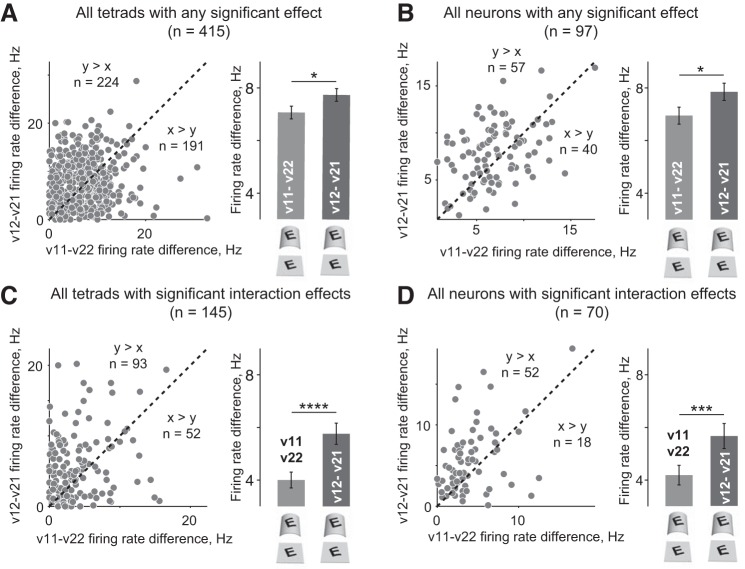
Tetradwise and neuronwise analyses. *A*: firing rate difference between v12 and v21 versions plotted against difference between v11 and v22 versions with each point representing an individual tetrad with a significant (main or interaction) effect (*n* = 415 tetrads). The dashed line is *y* = *x*, and the numbers on either side indicate the data points on either side. The bars on the right depict average firing rate differences with error bars indicating SE (same as Fig. 4*F*). Asterisks represent statistical significance (*P < 0.05, **P < 0.005, etc.) on a signed-rank test. *B*: firing rate difference between v12 and v21 plotted against difference between v11 and v22 with each point representing an individual neuron (i.e., the average across tetrads with a significant main/interaction for a given neuron; *n* = 97 neurons). All conventions are as in *A*. *C*: similar plot as in *B* except for tetrads with interaction effects (same data as Fig. 4*B*). *D*: similar plot as in *C* except for neurons with interaction effects.

In the above analyses, the main and interaction effects may have arisen within a small subset of neurons, which could have, in turn, resulted in an inflated statistical significance at the neuronal level. To verify that this was not the case, we repeated the above analyses after averaging the neural dissimilarity across tetrads recorded from each neuron. To this end, we first selected the neurons that showed a main or an interaction effect at the level of individual tetrads. For these neurons (*n* = 96), we first plotted the neural dissimilarity (average firing rate difference) for v11–v22 pairs against the v12–v21 pairs for each tetrad as before (Fig. 5*A*). We then plotted the same data after averaging the tetrads with a significant effect for each neuron, so that there is one data point per neuron—this too showed the same effect (Fig. 5*B*). The same was true on plotting firing rate differences for interaction effects across tetrads (Fig. 5*C*) or after averaging across tetrads for each neuron (Fig. 5*D*). Thus, even when the data are averaged across tetrads for a given neuron, neuronal responses show greater similarity across the perceptually equivalent stimuli (v11–v22), indicating that IT neurons decouple patterns from surfaces.

### Surfacewise Analysis of Interaction Effects

**Fig. 6.**
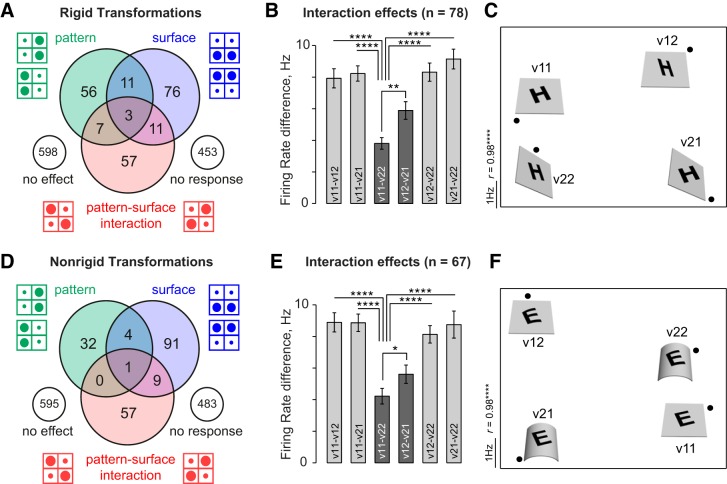
Interaction effects for rigid and nonrigid surface transformations. *A*: Venn diagram of main and interaction effects for tetrads involving rigid (tilt/rotate) transformations. *B*: neural dissimilarity for all pairs of stimuli for tetrads with rigid transformations. *C*: visualization of dissimilarity relations in *B* using multidimensional scaling. *D–F*: same as in *A–C* but for tetrads with nonrigid (convex/concave) transformations.

To confirm that the above surface-pattern interactions are present in both rigid and nonrigid surface/pattern transformations, we repeated the key comparison in the above analyses separately for rigid transformations (tilt/rotate: Fig. 6, *A*–*C*) and for nonrigid transformations (convex/concave: Fig. 6, *D–F*). We obtained similar results for both types of transformations—in other words, pattern-surface interactions tended to produce similar responses to congruent rigid or nonrigid transformations of patterns and surfaces.

### Do Surface-Pattern Interactions Tend to Co-occur Across Cells?

Do the pattern-surface interactions tend to occur within the same population of neurons? If this were true, a neuron that showed pattern-surface interactions for one set of patterns/surfaces should also show interaction effects for another independent set of patterns and surfaces. To investigate this issue, we performed a split-half analysis as follows. We divided the patterns and surfaces tested on each cell into two random groups of three patterns/two surfaces each (i.e., 6 tetrads per group). We then calculated the strength of the pattern-surface interaction as the absolute difference between the mean firing rate (50–250 ms after image onset) along one diagonal and the mean firing rate along the other diagonal. Next, we asked whether cells that show a large interaction effect strength for one group of patterns/surfaces would show a large interaction for the other group. A positive correlation would imply that the same cells show consistently larger or smaller interactions across patterns/surfaces. Indeed, this analysis yielded a weak but statistically significant correlation (median correlation: *r* = 0.41 across 1,000 random surface/pattern splits; this correlation was >0 in all splits). We obtained similar correlations for pattern and surface modulations as well (*r* = 0.42 for pattern; *r* = 0.36 for surface; both correlations were positive in all the splits). We conclude that pattern-surface interactions (as well as pattern and surface modulations) occur more consistently in some neurons than others, indicative of a single subpopulation that extracts patterns from surfaces.

### Mechanisms Underlying Pattern-Surface Interactions

**Fig. 7.**
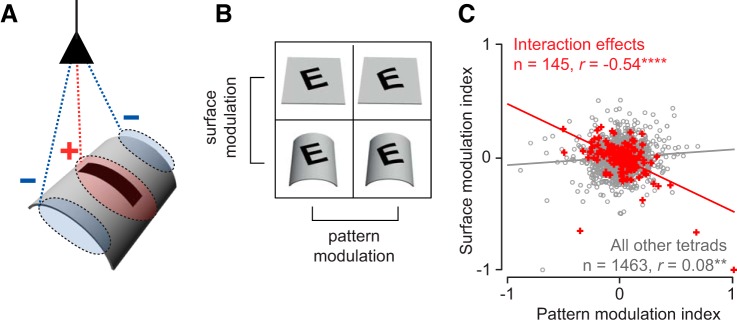
Mechanisms underlying surface-pattern interactions. *A*: schematic showing how surface-pattern interactions might arise. A neuron receives excitatory modulation from the central pattern but inhibitory modulation from the surrounding surface features. The inhibitory modulation results in surface features being factored out of pattern features. *B*: for each tetrad, we calculated the degree of response modulation for pattern changes (pattern modulation index) and for surface changes (surface modulation index). *C*: surface modulation index plotted against pattern modulation index, for tetrads with interaction effects (*n* = 145, red crosses) and all remaining tetrads (*n* = 1,463, gray circles).

How are these pattern-surface interactions computed? We reasoned that similar responses to congruent stimuli could arise if the response increases for a pattern change and decreases for an equivalent surface change, resulting in no net change in response when both pattern and surface change. This can be implemented by a neuron that receives excitatory modulation from pattern changes and inhibitory modulation from surface changes, or vice versa (Fig. 7*A*).

It is instructive to consider what happens in the simple case when excitatory and inhibitory influences combine linearly. Consider a neuron with a baseline response of *B* for the tetrad stimulus v11, that received an excitatory modulation of Δ across a pattern change and an equal but opposite (inhibitory) modulation of −Δ for a surface change. Then the responses to the four stimuli in the tetrad would be *B*, *B* + Δ, *B* − Δ, and *B*. While this would produce the desired effect (i.e., equal responses to v11 and v22), this linear summation would produce two main effects but not an interaction effect. The fact that we observed surface invariance only for interaction effects (Fig. 4*B*) but not for main effects (Fig. 4*D*) thus indicates a nonlinear summation of excitatory and inhibitory modulations.

To investigate this further in our data, we asked whether pattern and surface modulations were negatively correlated for tetrads with surface-pattern interactions. For each tetrad, we calculated a modulation index that measures how strongly the response was modulated by a surface change or a pattern change. The pattern modulation index was estimated as (*T* − *F*)/(*T* + *F*), where *F* is the average response to the flat pattern and *T* is the average response to the transformed pattern (Fig. 7*A*). The surface modulation index was calculated analogously. We then asked whether pattern modulation was correlated with the surface modulation across tetrads. Tetrads with pattern-surface interactions showed a significant negative correlation between the surface and pattern modulation indexes (*r* = −0.54, *P* < 0.00005, Fig. 7*C*). This correlation was present even upon removing the three outlier points with surface modulation close to −1 (*r* = −0.43, *P* < 0.00005). It was present for both rigid rotate/tilt transformations (*r* = −0.56, *P* < 0.00005) as well as for nonrigid (convex/concave) transformations (*r* = −0.51, *P* < 0.00005). In contrast, there was no such correlation across tetrads without these interactions, i.e., with main effects (*r* = 0.08, *P* = 0.001). Thus surface invariance arises through nonlinear interactions that produce opposite tuning for surface and pattern changes.

### Experiment 2: Impact of Surface Curvature on Pattern Tuning

In *experiment 1*, we have shown that a subpopulation of IT neurons exhibit surface-pattern interactions that decouple patterns from surfaces. However, there were two shortcomings of this experiment which we sought to address using a follow-up experiment. First, the surface-pattern interaction effects may be much stronger if the stimuli were matched to the preference of the neurons. Second, the fact that patterns and surfaces underwent congruent changes raises a potential concern: the curved E in Fig. 1*B*, for instance, may be decoupled from a curved surface only when its curvature is congruent with that of the surface. This could happen because both pattern and surface share features or alignment cues when both are flat and both are curved.

**Fig. 8.**
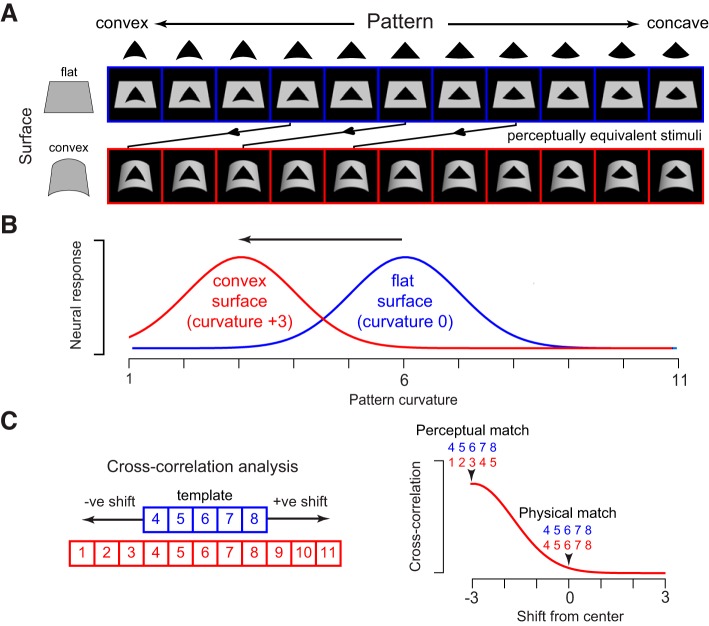
Design of *experiment 2*. *A*: stimuli used in *experiment 2*. Each pattern was parametrically varied from convex (left) to concave (right) in 11 regular steps and then overlaid either on a flat surface (top row) or on a convex surface (bottom row). The convex surface was generated to have a curvature that corresponded exactly to three levels of pattern curvature. As a result, each pattern variation on the flat surface is equivalent (through a nonrigid transformation) to a pattern variation on the convex surface that is shifted exactly by three steps to the left (arrows). *B*: hypothetical neuronal responses. A neuron that responds to the physical pattern regardless of the surrounding surface will show identical responses to the two rows. However, a surface-invariant neuron will shift its tuning by exactly three steps to produce equal responses to the congruent stimuli. Note that this tuning curve is depicted as Gaussian for simplicity but could be any arbitrary tuning curve without violating the essential logic. *C*: cross-correlation analysis and expected outcome. For each set of responses to pattern variations on flat and convex surfaces, we calculated the correlation between responses to patterns on the flat surface (patterns 4–8, in blue) and responses to shifted patterns on the convex surface, with shifts to the right denoted as positive by convention. If surface invariance arises through systematic shifts in pattern tuning, we predict that the response correlation will be maximum when the stimuli are aligned to be equivalent across nonrigid transformations (i.e., by a shift of −3).

To address these lacunae, we performed an additional experiment on a subset of the neurons in *experiment 1* that (during recording) appeared to show pattern-surface interactions (see methods). In this experiment (*experiment 2*), the preferred pattern of each neuron was bent parametrically such that it varied from being bent up (defined as concave) or bent down (defined as convex) in 11 equal parametric steps (Fig. 8*A*). These pattern variations were overlaid on either a flat surface or a curved surface that was produced by bending the flat surface by three curvature steps. The consequence of this design is that each pattern on a flat surface is equivalent (across a nonrigid transformation) to a pattern on a curved surface that is exactly three steps away (Fig. 8*A*). Consider then a hypothetical neuron with a Gaussian tuning with a peak centered at one of the pattern curvature levels (Fig. 8*B*). Note that this tuning curve is depicted as Gaussian for simplicity but could be any arbitrary tuning curve without violating the essential logic. We reasoned that this neuron should show a systematic shift in tuning for pattern curvature for patterns against a curved surface compared with patterns on a flat surface (Fig. 8*B*). To assess this possibility, we performed a cross-correlation analysis (Fig. 8*C*). We shifted the responses to pattern variations against a flat surface (*elements 4–8* in Fig. 8*C*) and calculated for each shift the correlation with the responses to the same patterns against a curved surface. If a neuron were selective only for the physical shape of the pattern regardless of the surface, this analysis would yield the best correlation for a shift of zero. On the other hand, if the neuron were factoring out the surface curvature from each pattern, the best correlation would occur for a shift of −3 (Fig. 8*C*).

**Fig. 9.**
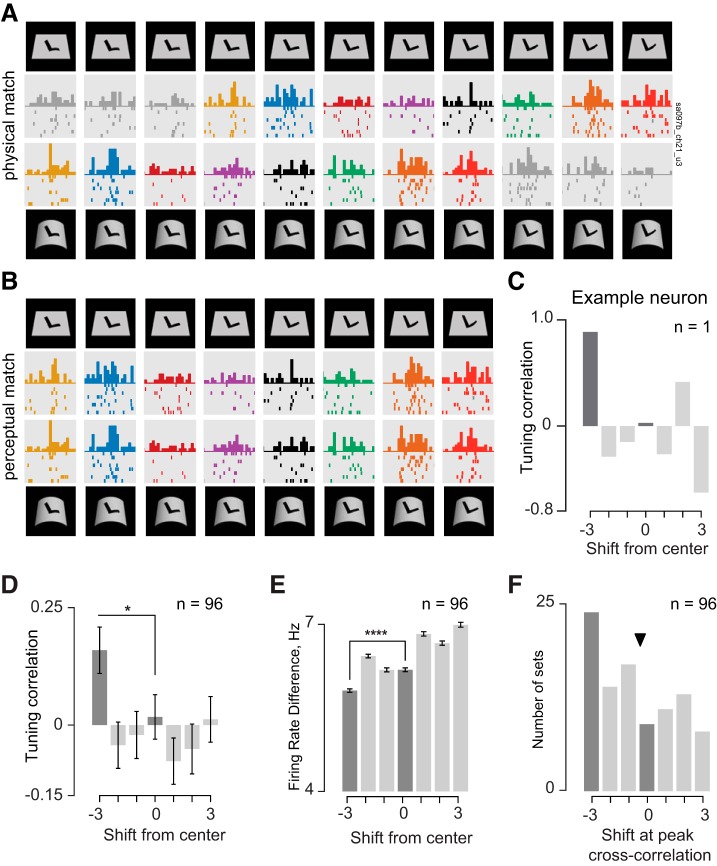
Surface context induces systematic shifts in tuning for pattern curvature. *A*: example responses of a single IT neuron to the pattern variations against flat and convex surfaces. Here, the patterns in each column are physically identical, but it can be seen that the responses are not the same. Raster plots with identical color represent stimuli in which the pattern and surface have undergone congruent nonrigid transformations. *B*: same responses as in *A* but now the stimuli are shifted to be nonrigidly equivalent. *C*: cross-correlation result for the same example neuron, showing that the tuning correlation is maximum when the responses to the flat surface are shifted to the left by three steps. *D*: average tuning correlation across all 96 sets of responses recorded from 58 neurons. The tuning correlation is maximum when responses are shifted by −3. The dark grey bars indicate the pattern-surface congruent (shift = −3) and pattern-identical conditions (shift = 0). Error bars indicate SE across the 96 response sets. *E*: average neuronal dissimilarity, calculated as the average firing rate difference between responses at each level of shift, showing the same result as in *D*. Error bars indicate the SE across the 96 sets of responses. *F*: distribution of the peak shift across all 96 response sets. The *x*-axis represents the shift level at which the cross-correlation between tuning curves attained a peak, and ▼ indicates the average shift across all sets (average shift = −0.4).

We tested each neuron on variations of at least 1 pattern, with the result that we obtained a total of 96 sets of responses from 58 IT neurons. The responses of an example IT neuron are shown in Fig. 9*A*. Here, the responses are aligned so that the patterns on the two surfaces are physically identical. This neuron responded differently to the same physical pattern presented against a curved and flat surface (Fig. 9*A*). However, strikingly, when its responses are shifted so that nonrigid equivalent stimuli are aligned together (i.e., for a shift of −3), its responses are much more similar (Fig. 9*B*). We confirmed this quantitatively using cross-correlation on the firing rates (100–200 ms after stimulus onset): responses to pattern variations were correlated best across the two surfaces when the flat surface responses were shifted by −3 (Fig. 9*C*). Thus this neuron shows a shift in tuning for pattern curvature against a curved surface compared with a flat surface, and this shift is exactly as expected if neurons were invariant to patterns across nonrigid transformations.

This effect was present across the 96 sets tested across the 58 neurons: the average tuning correlation for a shift of −3 was larger than the tuning correlation at all other shifts (*P* < 0.05, rank-sum test, Fig. 9*D*). We observed the same result using neuronal dissimilarity: for each shift in tuning, we calculated the average neuronal dissimilarity between the patterns on the flat surface and patterns on the curved surface. This too revealed that the nonrigid equivalent stimuli were the most similar (Fig. 9*E*). To further elucidate this shift in tuning, we calculated in each case the tuning shift that yielded the best cross-correlation. The largest number of instances had a preferred shift of −3 (*n* = 24 of 96 or 25%); these occurred across 21 of 56 neurons (i.e., 38%) (Fig. 9*F*).

We conclude that IT neurons show systematic shifts in tuning to pattern variations on a convex surface compared with a flat surface.

### Tuning Shifts for Concave Surfaces

Although the above analyses were performed on neuronal responses to patterns overlaid on flat and convex surfaces, we also tested neurons in this experiment on patterns overlaid on concave surfaces but did not obtain the same result as with convex surfaces. We present our analyses for concave surfaces below and discuss the possible reasons for this discrepancy.

According to our experimental design (Fig. 8), surface-invariant neurons should show a systematic shift that is opposite in direction when patterns are overlaid on concave surfaces compared with convex surfaces. Conversely, we expect no systematic changes in pattern curvature tuning in neurons that do not show surface-pattern interactions.

**Fig. 10.**
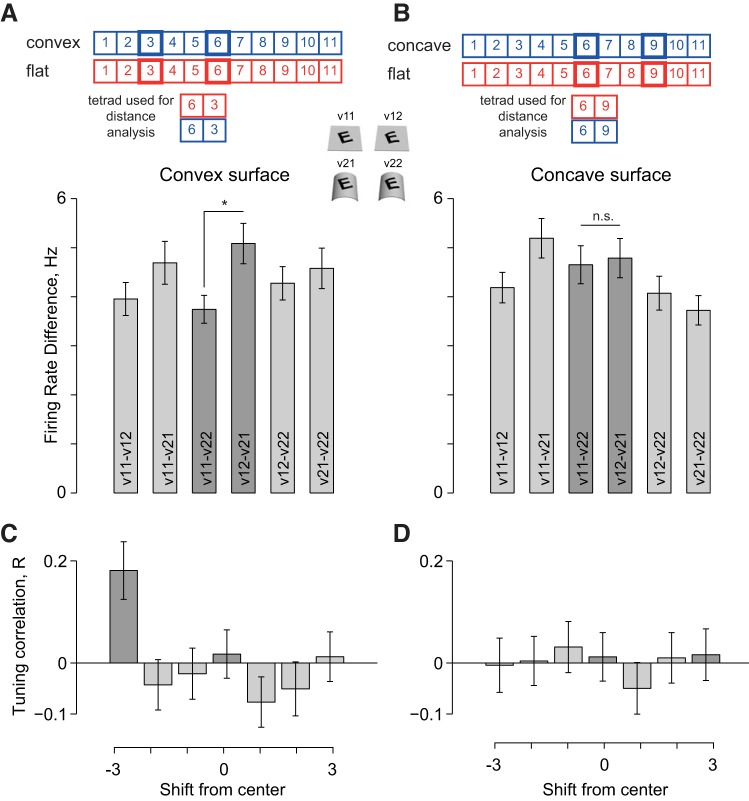
Tuning shift analysis for convex and concave surfaces. *A*: firing rate differences for all pairs of versions, for the tetrad containing perceptually matched stimuli (*top*) for the convex surface (*n* = 96 cases). It can be seen that the key comparison, i.e., v11–v22, is significantly smaller than v12–v21 (*P < 0.05 on a rank-sum test), indicating that surface-pattern interactions were prevalent for convex surfaces in the sampled population of neurons. *B*: similar plot as in *A* for concave surfaces. Here, we did not observe a statistically significant difference between v11–v22 and v12–v21 although the effect was in the correct direction. This implies that surface-pattern interactions were infrequent or weak for concave surfaces in the sampled population. *C*: average tuning correlation across all 96 sets of responses from 58 neurons for the convex surface (same plot as Fig. 9*D*). The tuning correlation is maximum when the responses are shifted by −3. The dark grey bars indicate the perceptually equivalent (shift −3) and the physically equivalent conditions (shift 0). Error bars indicate SE across the 96 sets. *D*: same as *C* for the concave surface showing no systematic shift in the responses.

We therefore set out to confirm whether neurons tested in *experiment 2* exhibit surface-pattern interactions in this experiment by selecting tetrads of stimuli identical to those tested in *experiment 1*. Specifically, we compared the firing rate difference between all pairs of versions (v11–v12, v11–v21, etc.) for the tetrad containing perceptually matched surface/pattern transformations for both the convex surface (Fig. 10*A*) and the concave surface (Fig. 10*B*). Across the population of neurons sampled, surface-pattern interactions were statistically significant for the convex surface (i.e., v11–v22 was smaller than v12–v21; Fig. 10*A*) but not for the concave surface (Fig. 10*B*). We then analyzed the neuronal responses to convex and concave surfaces for shifts in tuning for pattern curvature as before. We found systematic shifts in pattern curvature tuning for convex surfaces (Fig. 10*C*) but not for concave surfaces (Fig. 10*D*).

To summarize, for neurons that exhibit surface-pattern interactions (for convex surfaces) we observed systematic shifts in pattern tuning. For concave surfaces, these neurons showed neither the systematic pattern-surface interactions nor any systematic shifts in pattern tuning. Our conservative explanation of this finding is that neurons selected for further testing in *experiment 2* may have been chosen inadvertently when they exhibited interaction effects for convex but not for concave surfaces. The other possibility is that surface invariance is more prevalent for convex compared with concave surfaces. When we investigated this possibility in *experiment 1*, we did not find a greater incidence of surface-pattern interactions for convex (32 of 145 interactions) compared with concave surfaces (35 of 145 interactions). However, this finding is consistent with the representational bias observed in IT neurons for convex over concave surfaces (Vaziri and Connor 2016; Yamane et al. 2008) and in human lateral occipital complex (Haushofer et al. 2008). This bias, in turn, may reflect the geometric property that any closed surface must have more convex than concave surfaces. Testing these possibilities, however, will require a more systematic study of surface invariance for convex and concave surfaces.

## DISCUSSION

Here we have shown for the first time that IT neurons exhibit a novel form of invariance, which we term as surface invariance: they encode patterns after factoring out the surface on which it is embedded. We have shown that surface invariance is produced by nonlinear pattern-surface interactions (*experiment 1*) that produce systematic shifts in pattern tuning depending on the surface (*experiment 2*). These surface-invariant responses can potentially support complex perceptual inferences in which patterns have to be recognized across changes in the surface on which they are overlaid. Below we discuss our findings in the context of the existing literature.

### Relation to Nonlinear Interactions in IT Neurons

Our finding that IT neurons exhibit relatively few surface-pattern interactions is consistent with previous reports of weak interactions between color and shape in IT neurons (McMahon and Olson 2009), between spatially separated parts within an object (Sripati and Olson 2010b), and between the sizes of object parts (Vighneshvel and Arun 2015). It is also consistent with the presence of nonlinear interactions between surface and contour fragments observed in IT neurons (Brincat and Connor 2004, 2006; Yamane et al. 2008). However, we have also shown that the surface-pattern interactions, when they do occur, are extremely systematic and occur within the same set of neurons. This finding is reminiscent of our recent finding that part-size interactions result in sensitivity to relative size in IT neurons (Vighneshvel and Arun 2015). These two studies together indicate that nonlinear interactions between object features in IT neurons are systematic and serve specific perceptual goals. This is also consistent with the recent finding that V4 neurons encode normalized rather than absolute curvature (El-Shamayleh and Pasupathy 2016).

Our results also elucidate the mechanism by which patterns are decoupled from surfaces. We have shown that surface-pattern interactions lead to opposite nonlinear modulation for patterns and surfaces: this is reminiscent of a center-surround organization with antagonistic tuning between the pattern and the surrounding surface. Our finding that pattern curvature tuning is systematically biased when juxtaposed against a curved surface can be interpreted as a specific type of contextual modulation: the surface shape information in the immediate vicinity of a pattern must be factored out of the pattern representation itself.

### Relevance to Pattern Perception

We now discuss an important aspect of our study, namely, that our results are based on recording neuronal activity while monkeys performed a fixation task. Our results do not imply that the animals had a surface-invariant perception of the patterns—demonstrating this requires training the animals to report their percept and using perturbation techniques to establish a causal link. However, the fact that surface-pattern interactions are highly systematic and lead to surface invariance even in the absence of explicit training implies that surface invariance can arise in IT even through unsupervised learning. The observed frequency of interactions in this study may in fact be a lower bound on their incidence: they may be stronger and more widespread when animals engage in behaviors that require decoupling of patterns from surfaces.

Our study may also have underestimated the true frequency of interactions because we did not test each neuron with its preferred stimuli or scale the stimuli to lie within its receptive field. This could have resulted in more pattern modulation in neurons with receptive fields too small to sense surface changes and more surface main effects in neurons with receptive fields too large to be sensitive to subtle pattern changes. Thus we may have observed more frequent interactions if the stimuli were scaled to the receptive field size. We did not tailor stimuli to each neuron because our goal was to characterize how an entire neural population in IT represents a fixed set of stimuli. Moreover, the presence of a pattern-surface interaction by definition implies that the neuron is sensitive to both pattern and changes, which, in turn, implies that the stimulus is contained within the receptive field.

### Relation to Other Forms of Invariance

IT neurons are well known for their invariance to many identity-preserving transformations such as size, position, and viewpoint (Connor et al. 2007; Tanaka 1992). We have demonstrated a novel form of invariance in IT neurons, namely, that they are invariant to nonrigid transformations of surfaces. Our finding is concordant with a recent study from our laboratory where we have shown that a subset of IT neurons are sensitive to the relative size of items in a display (Vighneshvel and Arun 2015). Both surface invariance and relative size coding were subtle effects in that they were present only in a subset of IT neurons and were detected only using subtle but systematic shape variations. In both studies, many IT neurons were insensitive to these subtle shape variations suggesting that they encode gross features of patterns whereas clearly only a minority of cells are sensitive to subtle variations. Our results show that responses to subtle variations are highly systematic and reflect real-world regularities.

Taken together, observations of invariance in IT neurons raise the more general question: What family of image variations produce invariant responses in IT neurons or, for that matter, in perception? Recent studies have referred to these variations as “identity-preserving” transformations (DiCarlo and Cox 2007; Li et al. 2009; Rust and DiCarlo 2012; Sharpee et al. 2013; Zhivago and Arun 2016; Zoccolan et al. 2007), although this of course raises the more fundamental question: What is object identity? One compelling possibility is that identity is inferred when objects undergo slow and smooth spatiotemporal variations during natural experience (Li and DiCarlo 2008, 2010), but precisely how associations learned with specific objects generalize to all objects remains an important open question.

### Conclusions

Taken together, our findings add to a growing body of evidence that neuronal representations in IT neurons predict aspects of human perception both qualitatively (Rollenhagen and Olson 2000; Vighneshvel and Arun 2015) and quantitatively (Op de Beeck et al. 2001; Sripati and Olson 2010a; Zhivago and Arun 2014). Recent studies have shown that IT neurons are selective for surface shape independent of the disparity or texture cues that define the surface (Liu 2004; Orban 2011). Our findings complement this observation by showing that IT neurons are selective for patterns independent of the surface shape. These studies suggest that IT neurons can decouple surfaces from patterns and patterns from surfaces so as to produce independent representations of both.

## GRANTS

This research was funded by a Senior Research Fellowship from the Council of Scientific and Industrial Research (N. A. Ratan Murty) and an Intermediate Fellowship from the Wellcome Trust-DBT India Alliance, and the DBT-IISc partnership programme (S. P. Arun).

## DISCLOSURES

No conflicts of interest, financial or otherwise, are declared by the author(s).

## AUTHOR CONTRIBUTIONS

N.A.R.M. and S.P.A. conceived and designed research; N.A.R.M. and S.P.A. performed experiments; N.A.R.M. and S.P.A. analyzed data; N.A.R.M. and S.P.A. interpreted results of experiments; N.A.R.M. prepared figures; N.A.R.M. and S.P.A. drafted manuscript; N.A.R.M. and S.P.A. edited and revised manuscript; N.A.R.M. and S.P.A. approved final version of manuscript.
